# Whole‐Genome Sequencing Unravels Virulence and Antimicrobial Resistance Landscapes of Major Bovine Respiratory Disease Pathogens

**DOI:** 10.1155/vmi/8022936

**Published:** 2026-10-19

**Authors:** Kaiwen Yin, Shuying Liu, Yue Sun, Haiyan Dong, Kaifan Han, Wei Mao, Weiguang Zhou, Hongxia Zhao

**Affiliations:** ^1^ College of Veterinary Medicine Inner Mongolia Agricultural University Hohhot 010000 Inner Mongolia China imau.edu.cn; ^2^ Xing’an League Animal Disease Prevention and Control Center Xing’an League 137400 Inner Mongolia China; ^3^ Modern Farming (group) Corporation Hohhot China

**Keywords:** bovine respiratory diseases, *Mannheimia haemolytica*, *Mycoplasma bovis*, *Pasteurella multocida*, whole-genome sequencing

## Abstract

Bovine respiratory diseases are a seasonally highly prevalent infectious condition and cause great economic losses to the farming industry. In order to expand the genomic database information of bovine respiratory disease pathogens, the raw data were assembled and genome annotated using relevant bioinformatics analysis software to clarify the strain virulence, resistance gene carriage, and evolution of bovine‐origin *Pasteurella multocida*, *Mannheimia haemolytica*, and *Mycoplasma bovis*. Whole genome sequencing of *Pasteurella multocida* (Pm1), *Mannheimia haemolytica* (Mh1), and *Mycoplasma bovis* (Mb1) isolated from diseased material was carried out using Illumina HiSeq *X* and Oxford Nanopore sequencing platforms. The whole genome size of Pm1, Mh1, and Mb1 is 620,451, 2,718,449, and 1,006,140 base pairs (bp), and the GC content is 40.25%, 41.07%, and 29.29%, respectively. Additionally, in Pm1, Mh1, and Mb1, the Cluster of Orthologous Groups (COG) in the database has the largest number of annotated genes, which are the annotated Pm1 (2020), Mh1 (2058), and Mb1 (1063) genes. Whereas, the number of genes annotated in the Comprehensive Antibiotic Resistance Database (CARD) is the lowest, with 50, 97, and 5 genes. This study, through whole‐genome sequencing and annotation of Pm1, Mh1, and Mb1, has enriched the gene pool of bovine respiratory disease pathogens. It contributes to a more comprehensive understanding of the genomic structure, functional gene distribution, and evolutionary relationships of these pathogens. The findings can support precise diagnosis and personalized treatment of bovine respiratory diseases, as well as help to develop targeted use of antibiotics for farm animals, thereby reducing the occurrence and spread of drug resistance. This research holds significant importance for promoting the healthy development of the livestock industry and mitigating economic losses.

**Simple Summary**
 Bovine respiratory diseases are a big problem for animal farming and cause huge economic losses. To address this issue, this study expanded the genomic database information of bovine respiratory disease pathogens and revealed the virulence, antibiotic resistance gene carriage, and evolutionary relationships of *Pasteurella multocida*, *Mannheimia haemolytica*, and *Mycoplasma bovis*. The research utilized the Illumina HiSeq *X* and Oxford Nanopore sequencing platforms to conduct whole‐genome sequencing of these three pathogens. Through this technique, the study contributes to a more comprehensive understanding of their genomic structure, functional distribution, and evolutionary relationships. The findings provide support for the precise diagnosis and personalized treatment of bovine respiratory diseases, while also aiding in the development of targeted drug use strategies to reduce the emergence and spread of drug resistance.

## 1. Introduction

Bovine respiratory disease is one of the diseases that cause the most serious harm to the cattle breeding industry in the world [1]. According to Ozsvari L et al. [2], 65% of the diseases in the breeding industry are related to respiratory diseases. The incidence rate is between 30% and 80%, and the fatality rate can reach 10% to 35%. Due to differences in region, pasture environment, treatment methods, and others, there is a significant 25% variation in mortality rates. The annual economic losses caused by bovine respiratory diseases are as high as 30 billion U.S. dollars globally. Among them, in the United Kingdom, 2.1 million cattle suffer from respiratory diseases each year, with 178,000 deaths, and the mortality rate is 8.5% [3, 4].

The causes of bovine respiratory diseases are diverse and often caused by abnormal body reactions induced by drastic changes in factors such as transportation and weather conditions [5]. This leads to the invasion and massive proliferation of pathogens that reside in the bovine respiratory tract, primarily including two major categories: bacteria and viruses. Common bacterial pathogens include *Pasteurella multocida* (Pm1) and *Mannheimia haemolytica* (Mh1). Infections caused by these bacteria can further weaken the host’s immune system, triggering secondary infections by various pathogens [6]. It has been discovered that pathogens such as Pm and Mh1 have multiple subtypes. For example, Pm can be classified into five capsular serotypes, namely A, B, D, E, and F [7, 8], while Mh1 has as many as 12 serotypes [9]. Each different subtype of these pathogens exhibits varying infectivity and virulence factors (VFs), and their functions also differ [10].

Additionally, *Mycoplasma bovis* (Mb1) is also an important pathogen in bovine respiratory diseases. Mb1 often co‐infects with Pm1 and Mh1. Due to the unique cell‐wall‐free structure of Mb1, treatment becomes more challenging [11–13]. The limited range of available drugs further aggravates the infection levels of Pm1 and Mh1. These factors collectively impact the preventive efficacy of vaccines, making it difficult to effectively prevent the occurrence of bovine respiratory diseases [14].

Whole‐genome sequencing (WGS) holds significant advantages in terms of comprehensiveness, resolution, discovery of new genes, analysis of drug resistance, virulence, and evolutionary research [15]. The combination of Illumina’s high accuracy and high coverage with Nanopore’s long‐read capabilities can generate more complete and accurate genome assembly results [16].

Therefore, in this study, WGS analyses were carried out on the highly pathogenic and drug‐resistant Pm1, Mh1, and Mb1 isolates from the samples of sick cattle with respiratory diseases by using the Illumina HiSeq *X* and Oxford Nanopore sequencing platforms. This study has provided a reference basis for further exploring the drug resistance and pathogenicity of bovine respiratory tract pathogens and also offered data support for the gene diversity of the pathogens causing bovine respiratory diseases.

## 2. Materials and Methods

### 2.1. Strain Source

The animal ethical approval was obtained, and nasal swabs from cattle with severe respiratory disease and lung tissue from deceased cattle were collected between June 2022 and December 2023. A total of 84 samples were collected from the local farms located at Hohhot, Inner Mongolia, China. The cattle were kept under conventional indoor housing systems with natural ventilation and concrete flooring with straw bedding and were fed a total mixed ration (TMR) ad libitum. Water was available ad libitum via automatic drinkers. The farms maintained standard veterinary care protocols, including routine vaccination and deworming.

### 2.2. Isolation, Selection, and DNA Extraction

The isolated Pm1, Mh1, and Mb1 were subjected to in vitro drug susceptibility testing. The disk diffusion method was used to determine the drug resistance profiles of Pm1 and Mh1 isolates, while the microdilution method was employed for Mb1 isolates. Additionally, the modified Karber’s (KB) method was used to determine the median lethal dose (LD50) of Pm1 and Mh1 isolates. For LD50 determination, SPF‐grade Kunming mice (18–22 g, male and female equally represented) were randomly divided into groups of 10 animals each. Ten‐fold serial dilutions of bacterial suspensions were prepared, and each mouse was intraperitoneally injected with 0.2 mL of the corresponding bacterial suspension. Mortality was monitored continuously for 5 days postinfection, and the LD50 values were subsequently calculated using the modified KB method. The presence of resistance and virulence genes in the isolates was identified using the PCR method. Each strain of Pm1, Mh1, and Mb1, which exhibited the strongest virulence and highest resistance rates, was selected. The Pm1 and Mh1 were inoculated into tryptic soy broth (TSB) medium containing 5% bovine serum and brain–heart infusion (BHI) medium at 37°C for 12–26 h. Mb1 was inoculated into a pleuropneumonia‐like organisms (PPLOs) liquid medium for 7–10 days. Bacterial genomic DNA was extracted according to the instructions of the Bacterial Genomic DNA Extraction Kit (TIANGEN BIOTECH [BEIJING] CO., LTD.) and stored at −20°C.

### 2.3. Genome Sequencing Analysis

The extracted DNA from the isolated bacteria was subjected to Nanodrop detection, with an A260/A230 ratio ranging between 1.8 and 2.0. The concentration measured by Qubit was ≥ 50 ng/μL. Further, genomic DNA that passed the quality check was sent to Shanghai OE Biotech Co., Ltd. for sequencing. A 1D library was constructed, and the PromethION sequencer was utilized to perform single‐molecule real‐time sequencing on the DNA, yielding raw sequencing data. The raw data underwent quality control, with low‐quality and excessively short reads being filtered out. The filtered reads were then subjected to de novo assembly, and the assembled draft genome was error‐corrected. Subsequently, the predicted gene sequences were employed for genomic structure analysis and functional annotation of the genome. Gene prediction for bacteria was performed using Prodigal‐2.6.2 software; tRNAs contained in the genome were predicted using tRNAscan‐SE 2.0 software; rRNAs contained in the genome were predicted using Barrnap 0.7 software; clusters of regularly spaced short palindromic repeats were predicted using MinCED 0.4.2 software; gene islands present in the genome were predicted using the http://www.pathogenomics.sfu.ca/islandviewer/web site; and prophages in the genome were predicted using the https://github.com/linsalrob/PhiSpy/web site. The Diamond software was used to annotate the gene functions of the whole genome sequence with the protein‐coding sequences of the predicted genes in the Cluster of Orthologous Groups (COG) of proteins, Gene Ontology (GO), Kyoto Encyclopedia of Genes and Genomes (KEGG), and Carbohydrate‐Active enZYmes (CAZy) databases; analyzed the pathogenicity and drug resistance in the Pathogen Host Interactions (PHI), Virulence Factors of Pathogenic Bacteria (VFPB), and Comprehensive Antibiotic Research Database (CARD). We selected the annotation results with an expected value (e‐value) less than 1 × 10^−10^. The links to each functional database are shown in Table 1.

**TABLE 1:** Functional database links.

Database name	Database link
COG	https://www.ncbi.nlm.nih.gov/COG/
GO	https://www.ncbi.nlm.nih.gov/GO/
KEGG	http://www.genome.jp/kegg/pathway.html/
CAZy	http://www.cazy.org/
PHI	http://www.phi-base.org/
VFDB	http://www.mgc.ac.cn/VFs/main.htm/
CARD	https://card.mcmaster.ca/

## 3. Result

### 3.1. Antibiotic Resistance Profile of the Isolates

Pm1, Mh1, and Mb1 are among the isolates that exhibit strong resistance and carry multiple resistance genes. According to the KB method, Pm1 shows resistance to penicillin G, amoxicillin, gentamicin, kanamycin, amikacin, streptomycin, lincomycin, and clindamycin. PCR identification reveals that Pm1 carries the resistance genes *strA*, *strB*, *qnrD*, *blaROB-1*, *tetB*, *gyrA*, *gyrB*, *parC*, and *parE*. Mh1 is resistant to penicillin G, amoxicillin, lincomycin, clindamycin, and erythromycin and carries the resistance genes *gyrA*, *gyrB*, *parC*, and *strA*. The MIC of Mb1 to various drugs was determined by the microdilution method, showing resistance only to enrofloxacin but intermediate resistance to tilmicosin, tulathromycin, erythromycin, azithromycin, sarafloxacin, ciprofloxacin, streptomycin sulfate, amikacin, and tetracycline. Mb1 carries the resistance genes *gyrA*, *gyrB*, *parC*, *parE*, *rrs3*, and *rrs4*; the results are shown in Table 2.

**TABLE 2:** Detection of drug resistance and virulence genes in Pm1, Mh1, and Mb1.

Strain	Drug resistance phenotype	Drug resistance gene	Virulence gene
Pm1	Penicillin G, amoxicillin, gentamicin, kanamycin, amikacin, streptomycin, lincomycin, clindamycin	*strA, strB, qnrD, blaROB-1, tetB, gyrA, gyrB, parC, parE*	*exbB*, *exbD*, *tonB*, *hgbB*, *fur*, *ptfA*, *fimA*, *hsf2*, *pfhA*, *tadD*, *ompA*, *ompH*, *oma87*, *plpE*, *psl*, *tbpA*, *sodC*, *nanB*, *pmHAS*
Mh1	Penicillin G, amoxicillin, lincomycin, clindamycin, erythromycin	*gyrA, gyrB, parC, strA*	*lktA*, *lktC*, *gs60*, *adh*, *tbpB*, *maA*
Mb1	Enrofloxacin	*gyrA, gyrB, parC, parE, rrs3, rrs4*	*VspX*, *VspY2*, *VpmA*, *P81*

### 3.2. Virulence Profile of the Isolates

The LD50 of Pm1 is 12 CFU·mL^−1^ and it carries the virulence genes *exbB*, *exbD*, *tonB*, *hgbB*, *fur*, *ptfA*, *fimA*, *hsf*2, *pfhA*, *tadD*, *ompA*, *ompH*, *oma*87, *plpE*, *psl*, *tbpA*, *sodC*, *nanB*, and *pmHAS*. The LD50 of Mh1 is 7.5 × 10^3^ CFU·mL^−1^, and it carries the virulence genes *lktA*, *lktC*, *gs60*, *adh*, *tbpB*, and *nmaA*. While Mb1 carries the virulence genes *VspX*, *VspY2*, *VpmA*, and *P81*, the results are shown in Table 2.

### 3.3. Genome Structure Analysis of Isolates

The Pm1, Mh1, and Mb1 strains isolated clinically were subjected to WGS. According to the sequencing results, the full‐length whole‐genome sequence of the Pm1 strain is 2,620,451 bp, with a GC content of 40.25%. It has 2532 protein‐coding genes, containing 60 tRNAs, 4 rRNAs, 1 tmRNA, 7 CRISPRs, 3 genomic islands, 9 simple tandem repeats, and no prophages. The full‐length whole‐genome sequence of the Mh1 strain is 2,718,449 bp, with a GC content of 41.07%. It has 2763 protein‐coding genes, containing 65 tRNAs, 5 rRNAs, 1 tmRNA, 1 CRISPR, 7 genomic islands, 16 simple tandem repeats, and no prophages. The full‐length whole‐genome sequence of the Mb1 strain is 1,006,140 bp, with a GC content of 29.29%. It has 1715 protein‐coding genes, containing 34 tRNAs and 11 genomic islands, and no CRISPR sequences or prophages were found. The data for Pm1 and Mh1 are similar, but Mb1 is significantly different from them, with a whole‐genome sequence only about one‐third the length of Pm1 and Mh1 and a GC content notably lower than that of Pm1 and Mh1. Additionally, Mb1 has fewer protein‐coding genes, suggesting that its genomic structure and function may differ considerably from those of Pm1 and Mh1. Furthermore, no prophages were detected in Pm1, Mh1, or Mb1.

### 3.4. Functional Annotation of Strain Genomes

The results of genome functional annotations for Pm1, Mh1, and Mb1 are shown in Table 3. Among them, the COG database with the largest number of annotated genes, which annotated 2020, 2058, and 1063 genes, respectively, while the number of genes annotated in the CARD is the least, with Pm1 (50), Mh1 (97) and Mb1 (5) genes. These results indicate that there are significant differences in the distribution of functional genes among the Pm1, Mh1, and Mb1 strains, particularly in the number of genes related to antibiotic resistance. The number of annotated genes in the Mb1 strain is significantly lower than that in Pm1 and Mh1.

**TABLE 3:** Overview of functional annotations of the isolated bacteria genome.

Database type	Number of coding genes		
	Pm1	Mh1	Mb1
COG	2020	2058	1063
GO	1805	1850	612
KEGG	1659	1599	934
CAZy	153	154	31
PHI	484	484	125
VFDB	220	233	46
CARD	50	97	5

#### 3.4.1. COG Database Annotation

Compare the protein‐coding genes of Pm1, Mh1, and Mb1 with the COG database. Among them, Pm1 has a total of 2020 genes annotated with 21 functions, including 169 genes encoding with amino acid transport and metabolism, 166 genes encoding with carbohydrate transport and metabolism, 164 genes encoding with translation, ribosomal structure, and biosynthesis, 150 genes encoding with cell wall/membrane/envelope biosynthesis, and 369 genes with unknown functions (Figure 1A). Mh1 has a total of 2058 genes annotated with 21 functions, including 165 genes encoding with amino acid transport and metabolism, 165 genes encoding with carbohydrate transport and metabolism, 159 genes with translation, ribosomal structure, and biosynthesis, and 147 genes encoding with cell wall/membrane/envelope biosynthesis functions (Figure 1B). Mb1 has a total of 1063 genes annotated with 18 functions, including 56 genes encoding with amino acid transport and metabolism, 78 genes encoding with carbohydrate transport, 178 genes encoding with translation, ribosomal structure, and biogenesis, 262 genes encoding with replication, recombination, and repair, and 15 genes encoding with cell wall/membrane/envelope and biogenesis functions (Figure 1C). A comparison of the COG databases of Pm1, Mh1, and Mb1 revealed significant differences in the distribution of functional genes among these strains. The number of annotated genes in Pm1 and Mh1 is similar, and their functional categories are relatively comprehensive, covering core functions such as amino acid transport and metabolism, carbohydrate transport and metabolism, translation and ribosome structure biogenesis, and cell wall/membrane/envelope biogenesis. In contrast, Mb1 has fewer annotated genes, with a higher number of genes related to replication, recombination, and repair.

**FIGURE 1:**
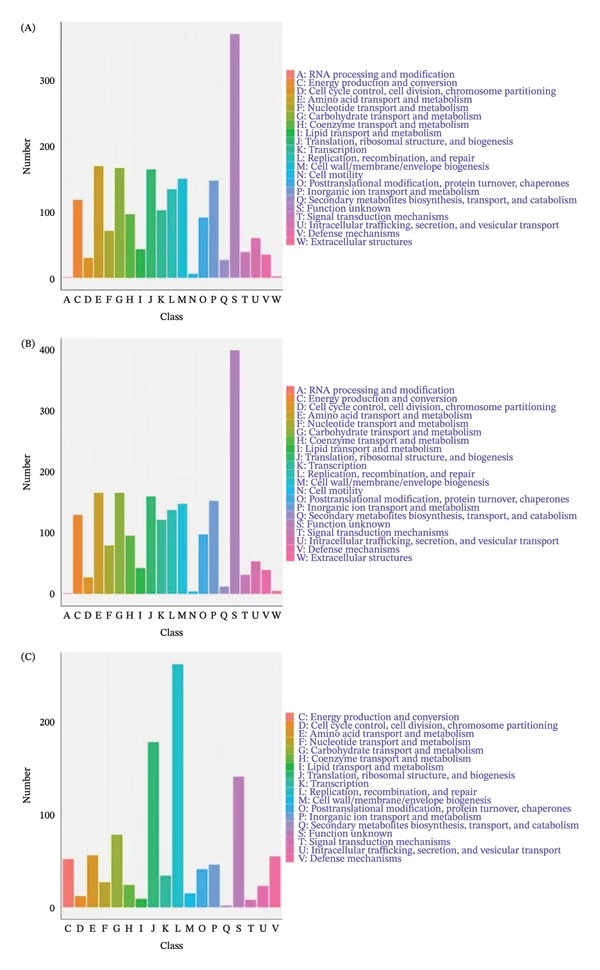
Statistical chart of COG annotation classification of genes.

The abscissa is the classification of COG; the ordinate is the number of genes.

#### 3.4.2. GO Database Annotation

Compare the protein‐coding genes of Pm1, Mh1, and Mb1 with the GO database. A total of 1805 genes were annotated for Pm1. Among them, the cellular and metabolic processes in the biological process are the two pathways with the highest gene enrichment levels, with 1000 and 935 genes annotated, respectively; the cell and cell part in the cellular component are the two pathways with the highest gene enrichment levels, with 1204 and 1200 genes annotated, respectively; and the catalytic activity and binding molecular function are the two pathways with the highest gene enrichment levels, with 1082 and 995 genes annotated, respectively (Figure 2A). A total of 1850 genes were annotated for Mh1. Among them, the cellular and metabolic processes in the biological process are the two pathways with the highest gene enrichment levels, with 1007 and 953 genes annotated, respectively; the cell and cell part in the cellular component are the two pathways with the highest gene enrichment levels, with 1186 and 1181 genes annotated, respectively; and the catalytic activity and binding molecular function are the two pathways with the highest gene enrichment levels, with 1081 and 1042 genes annotated, respectively (Figure 2B). A total of 612 genes were annotated for Mb1. Among them, the cellular processes and metabolic processes in the biological process are the two pathways with the highest gene enrichment levels, with 403 and 397 genes annotated, respectively; the cell and cell part in the cellular component are the two pathways with the highest gene enrichment levels, with 421 and 416 genes annotated, respectively; and the catalytic activity and binding molecular function are the two pathways with the highest gene enrichment levels, with 483 and 457 genes annotated, respectively (Figure 2C). The number of annotated genes in Pm1 and Mh1 is similar, and in the three major categories of biological processes, cellular components, and molecular functions, pathways such as cellular processes, metabolic processes, cell and cell parts, and catalytic activity, and there is a high level of gene enrichment binding. This indicates that Pm1 and Mh1 exhibit high functional diversity in cellular metabolism, structural maintenance, and molecular functions. In contrast, Mb1 has only 612 annotated genes, yet it still demonstrates high gene enrichment levels in the same major pathways, particularly in catalytic activity and binding within the molecular function category.

FIGURE 2Statistical chart of GO annotation classification of genes.

**Figure:**
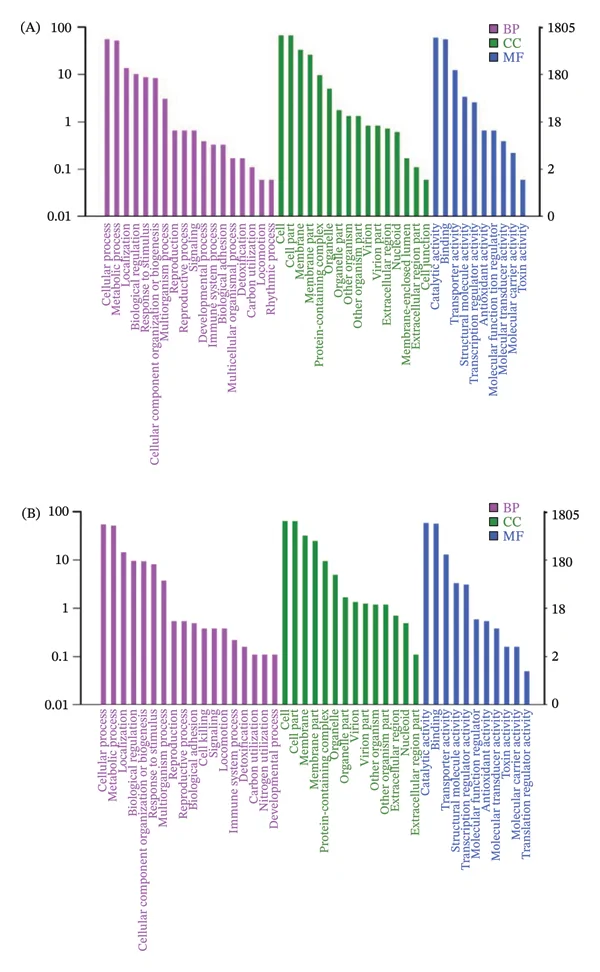

**Figure:**
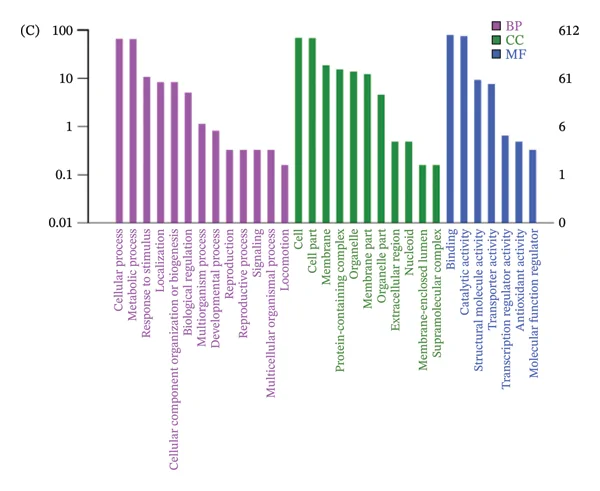

The abscissa is the classification of GO; on the left side of the ordinate is the percentage of the number of genes, and on the right side is the number of genes.

#### 3.4.3. KEGG Database Annotation

Compare the protein‐coding genes of Pm1, Mh1, and Mb1 with the KEGG database. Pm1 was annotated to a total of 1659 genes, among which the genes in the metabolic pathway had the highest enrichment degree, with 1129 genes annotated. Among them, the genes involved in carbohydrate metabolism were the most numerous, with 268 genes annotated, followed by the genes involved in amino acid metabolism and membrane transport, both annotated to 165 genes (Figure 3A). Mh1 was annotated to a total of 1599 genes, among which the genes in metabolism had the highest enrichment degree, with 1063 genes annotated. Among them, the genes involved in carbohydrate metabolism were the most numerous, with 265 genes annotated, followed by the global and overview maps and membrane transport genes, which were annotated to 207 and 176 genes, respectively (Figure 3B). Mb1 was annotated to a total of 934 genes, among which the genes in metabolism had the highest enrichment degree, with 437 genes annotated. The genes involved in carbohydrate metabolism were the most numerous, with 136 genes annotated. The genes involved in translation were annotated to 135 genes, followed by the replication and repair and membrane transport genes, which were annotated to 107 and 91 genes, respectively (Figure 3C). Pm1, Mh1, and Mb1 all exhibit high levels of gene enrichment in metabolic pathways, particularly showing significant functional diversity in core metabolic pathways such as carbohydrate metabolism, amino acid metabolism, and membrane transport. The number of annotated genes in Pm1 and Mh1 is similar, and both have a high number of genes involved in pathways such as carbohydrate metabolism, amino acid metabolism, and membrane transport. Although Mb1 has fewer annotated genes, it still demonstrates high enrichment levels in carbohydrate metabolism and translation‐related genes, with a notable number of annotations in replication and repair as well as membrane transport genes.

FIGURE 3Statistical chart of KEGG annotation classification of genes.

**Figure:**
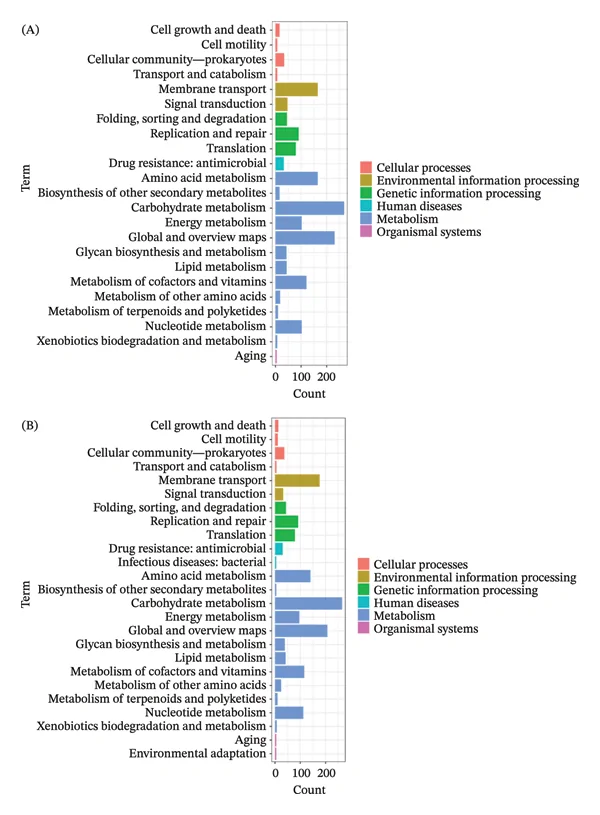

**Figure:**
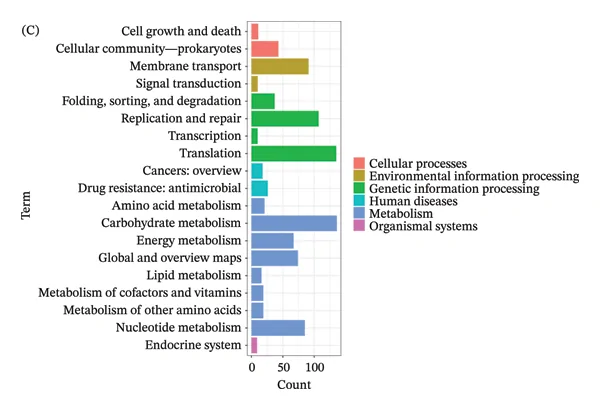

The abscissa is the number of genes; the ordinate is the classification of KEGG.

#### 3.4.4. CAZy Database Annotation

Compare the protein‐coding genes of the Pm1, Mh1, and Mb1 strains with the CAZy database. A total of 153 genes were annotated for Pm1, including 62 glycosyl transferases, 43 glycoside hydrolases, 19 auxiliary activities, 16 carbohydrate esterases, 7 polysaccharide lyases, and 6 carbohydrate‐binding modules (Figure 4A). A total of 154 genes were annotated for Mh1, including 63 glycosyl transferases, 42 glycoside hydrolases, 21 auxiliary activities, 18 carbohydrate esterases, 4 polysaccharide lyases, and 6 carbohydrate‐binding modules (Figure 4B). A total of 31 genes were annotated for Mb1, including 8 auxiliary activities, 6 carbohydrate esterases, 6 glycoside hydrolases, 8 glycosyl transferases, 1 carbohydrate‐binding module, and 2 polysaccharide lyases (Figure 4C).

**FIGURE 4:**
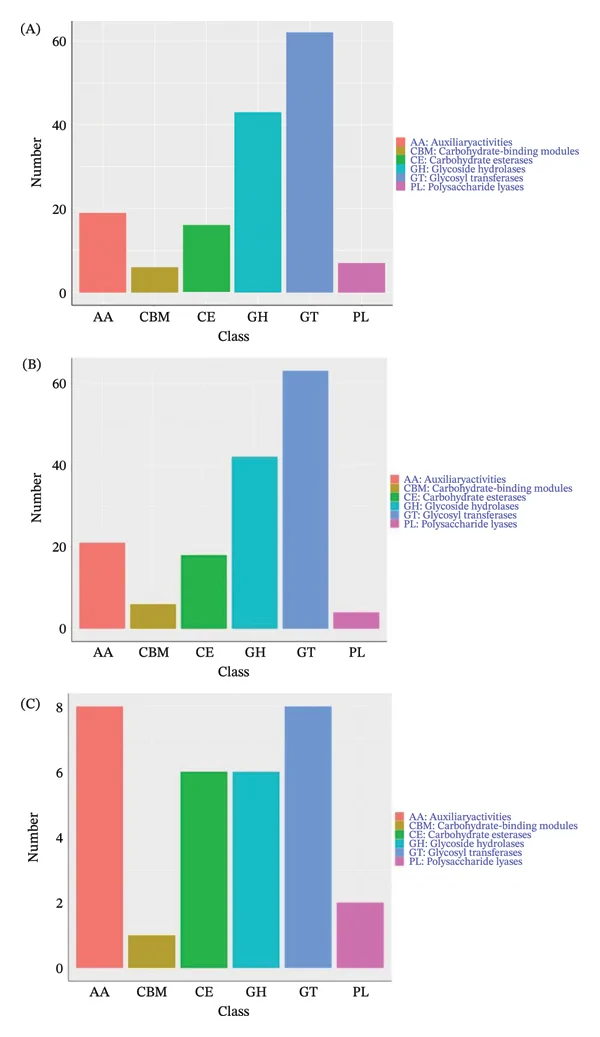
Statistical chart of CAZy annotation classification of genes.

The abscissa is the classification of CAZy; the ordinate is the number of genes.

#### 3.4.5. PHI Database Annotation

After comparing the protein‐coding genes of the Pm1, Mh1, and Mb1 strains with the PHI database, it was found that phenotypic mutations occurred in all of them. Among them, a total of 484 genes were annotated for Pm1. After mutation, the virulence of 31 genes was enhanced, that of 296 genes was weakened, 7 genes became lethal after mutation, 34 genes made the strain lose its pathogenicity after mutation, 108 genes were unaffected after mutation, 4 genes mutated into effectors, 2 genes had chemical resistance after mutation, and 2 genes had chemical sensitivity after mutation. A total of 484 genes were annotated for Mh1. After mutation, the virulence of 35 genes was enhanced, that of 324 genes was weakened, 7 genes became lethal after mutation, 55 genes made the strain lose its pathogenicity after mutation, 127 genes were unaffected after mutation, 3 genes mutated into effectors, 4 genes had chemical resistance after mutation, and 4 genes had chemical sensitivity after mutation. A total of 125 genes were annotated for Mb1. After mutation, the virulence of 11 genes was enhanced, that of 95 genes was weakened, 11 genes became lethal after mutation, 10 genes made the strain lose its pathogenicity after mutation, 13 genes had unaffected pathogenicity after mutation, 2 genes mutated into effectors, 3 genes had resistance to chemical, and 3 genes had sensitivity to chemical. Pm1, Mh1, and Mb1 all exhibit significant phenotypic mutations, which have varying degrees of impact on their virulence, pathogenicity, and chemical sensitivity. The number of annotated genes in Pm1 and Mh1 are the same, and they show similar trends after mutation: The virulence of most genes is reduced, the virulence of some genes is enhanced, and a small number of genes become lethal or cause the strain to lose pathogenicity after mutation. Although Mb1 has fewer annotated genes, its mutation pattern is similar to that of Pm1 and Mh1.

#### 3.4.6. VFDB Database Annotation

Compare the protein‐coding genes of the Pm1, Mh1, and Mb1 strains with the VFDB database. For Pm1, a total of 220 virulence genes were annotated. Among them, the largest number of genes were annotated to the VF LOS, with a total of 45 genes. Secondly, 28 genes were annotated to the capsule. In addition, genes such as *lpxA, glmU,* and *acpXL* related to LPS were also annotated. The relevant results are shown in Table 4. For Mh1, a total of 233 virulence genes were annotated. Among them, there were 36 genes for LOS at most, followed by 16 genes annotated to the capsule. In addition, genes such as *barB, basJ, bauE, bauC*, and *bauD* related to the acinetobactin gene were also annotated. The relevant results are shown in Table 5. For Mb1, a total of 46 virulence genes were annotated. The relevant results are shown in Table 6.

**TABLE 4:** Major virulence factors in the genome of Pm1 strains.

Virulence factor	Associated genes	Capability
Capsule	*ctrC*, *bexA*, *bexD′*, *bexC′*, *hcsA′*, *hcsB′*, *ugd*, *cpsE*, *pgi*, *rpe*, *wcbN*, *wzt2*, *cpsA*, *uppS*, *cpsB*, *cdsA*, *gndA*, *cap8E*, *cap8F*, *cap8G*, *cps4F*, *tviB*, *ctrC*	Immune modulation
LPS	*wbPm1*, *bplF*, *lpxA*, *glmU*, *acpXL*, *or fM*, *kdtB*, *wbuZ*, *hisH2*	
LOS	*rfaD*, *gmhA*, *kfiC*, *lgtA*, *neuA*, *galE*, *msbA*, *kpsF*, *kdsA*, *galU*, *yhbX*, *lpxH*, *lpxK*, *msbA*, *kdsB*, *manB*, *yhxB*, *kdkA*, *lgtF*, *kdtA*, *lic3A*, *lpcA*, *opsX*, *rfaC*, *waaQ*, *rffG*, *lsgA*, *lsgD*, *lsgB*, *lsgE*, *lsgF*, *lgtC*, *htrB*, *rfaE*, *wlaN*, *lpxB*, *lpxA*, *wecA*	
Alginate	*algU*, *algC*, *mucD*, *algW*, *mucP*	Biofilm
BopD	*bopD*	
AdeFGH efflux pump	*adeG*	
AI‐2	*luxS*	
VPS	*vpsG*, *vpsU*	
EF‐Tu	*tufA*	Adherence
Type IV pili	*pilR*, *vfr*, *pilL*, *tapQ*, *tapC*, *pilF*, *pilB*	
FHA	*fhaB*, *fhaC*	
Type 1 fimbriae	*fimE*	
RTX protein	*frpA*	
Hsp60	*htpB*	
Fur	*fur*	Iron uptake
Hemolysin	*hlyB*, *cesC*	Exotoxin
LpeA	*lpeA*	Invasion
OmpA	*OmpA*	
Polar flagella	*flhF*, *flmH*, *ylxH*, *eptC*	Motility

**TABLE 5:** Major virulence factors in the genome of Mh1 strains.

Virulence factor	Associated genes	Capability
Capsule	*wcbN*, *cap8O*, *hcsB′*, *cps4I*, *hcsA′*, *bexB′*, *bexC′*, *bexD′*, *pgi*, *bexA*, *cpsA/uppS*, *FTT_RS04105*, *gndA*, *rpe*, *Cj1437c*, *cpsB/cdsA*	Immune modulation
LPS	*kdtB*, *bplF*, *hisH2*, *LPG_RS03780*, *wbuZ*, *lpxA/glmU*	
LOS	*galU*, *or fM*, *lpxA*, *gmhA*, *galE*, *lpxK*, *kdtA*, *wecA*, *gmhA/lpcA*, *manB/yhxB*, *msbA*, *kdsB*, *wlaN*, *lpxH*, *kpsF*, *lpsA*, *kdkA*, *rfaF*, *lgtA*, *kdsA*, *rfaD*, *galU*, *lgtF*, *waaQ*, *htrB*, *rffG*, *lsgF*, *lpxB*, *opsX/rfaC*, *lsgE*, *rfaE*, *lsgA*, *lsgB*, *lsgD*, *yhbX*	
PDIM	*ppsC*, *ppsC*, *ddrA*, *Rv2952*	
Acinetobactin	*barB*, *basJ*, *bauE*, *bauC*, *bauD*	Nutritional/Metabolic factor
CHU	*chuX*, *chuU*, *chuV*, *chuT*, *chuW*	
Pyoverdine	*fpvA*, *ptxR*, *pvdH*, *PA2383*	
CcmC	*ccmA*, *ccmC*, *ccmE*, *ccmF*	
Biotin synthesis	*bioA*, *bioB*, *bioD*, *bioF*	
T4SS secreted effectors	*coxFIC1*, *CBU_1566*, *CBU_1434*	Effector delivery system
TTSS secreted effectors	*glgA*, *CT_473*, *glgX*	
Eps T2SS	*epsD*	
Type VII secretion system	*essC*	
TTSS	*cdsN*	
IlpA	*IlpA*	Adherence
Type IV pili	*pilR*, *vfr*, *pilL*, *LPG_RS04900*, *pilC*, *pilF*, *pilS*	
Ata	*ata*	
EF‐Tu	*tufA*	
FHA	*fhaB*	
Curli fibers	*cgsD*	
Hemolysin	*hlyA*, *hlyB*, *hlyC*, *hlyD*	Exotoxin
Colibactin	*clbI*, *clbG*	
Phospholipase A2	*par2*	
Cya	*cyaB*	
Alginate	*algC*, *mucP*, *algW*, *algU*, *mucD*	Biofilm
K1 capsule	*neuA*, *neuB*, *neuC*, *neuD*	Invasion
OmpA	*OmpA*	
Polar flagella	*flhF*, *flmH*	Motility
Flagella	*ylxH*, *flhF*, *pdxA*	

**TABLE 6:** Major virulence factors in the genome of Mb1 strains.

Virulence factor	Associated genes	Capability
Cereulide	*cesC*	Exotoxin
Cytolysin	*cylB*	
Cya	*cyab*	Effector delivery system
Hemolysin	*hlyB*	
MARTX	*rtxB*	
T4SS	*coxFIC1*, *cylG*	
HSI‐I	*ppkA*	
TTSS	*cdsN*	
LOS	*manB/yhxB*, *msbA*	Immune modulation
AdsA	*adsA*	
LPS	*KdtB*, *KP1_RS17220*,	
Capsule	*Rpe*, *hasC*, *Cps*, *J FTT_RS04120*	
Pyochelin	*pchH*	Nutritional/Metabolic factor
MgtBC	*mgtB*	
Sal	*iroC*	
LplA1	*lplA1*	
Chu	*chuV*	
MsrAB	*msrA/B*	Stress survival
ClpC	*clpC*	
EF‐Tu	*tufA*	Adherence
Type IV pili	*rpoS*	
TlyC	*tlyC*	Exoenzyme
SigA	*sigA/rpoV*	Regulation
K1 capsule	*kpsT*	Invasion
Flagella	*fliI*	Motility
Dispersin	*aatC*	Others

*Note:* Genes annotated by VFDB but considered likely annotation artifacts based on read coverage and assembly quality analysis; not validated as genuine *M. bovis* virulence factors.

#### 3.4.7. CARD Database Annotation

Compare the protein‐coding genes of the Pm1, Mh1, and Mb1 strains with the CARD database. For Pm1, a total of 50 resistance genes were annotated, including tetracycline resistance genes such as *tet (35), tetT, tetA (60), tetA (48), tet (W/N/W), tet (H), tet32, tetB (p), β*‐lactam resistance genes like *H-NS, cpxA, patA, patB, evgA, evgS, mecD, mupB,* aminoglycoside resistance genes including *vatB, oleC, clbA*, macrolide resistance genes such as *H-NS, ErmQ, CRP, TaeA,* and *mel*, sulfonamide resistance genes like *cpxA, sul4, dfrA3,* and fluoroquinolone resistance genes such as *mupA*, polypeptide resistance genes including *basS, eptA, arnA, vanTG, vanHM, vanRL, vanHA, vanRO, vanRE, vanI, ugd, bcrA*, *NmcR*, and nitroimidazole resistance genes like msbA, lincosamide resistance genes such as *lmrC, lmrD*, and chloramphenicol resistance genes including *poxtA, optrA*, and *cmx,* as well as multidrug efflux pump‐related genes like *macA, macB, mexW, mtrA,* and *qacH*. For Mh1, a total of 97 resistance genes were annotated, including tetracycline resistance genes such as *tetA (48)/(60), tetB (60), tet32/(35)/(43), tetT/(W/N/W), otr (A)*, sulfonamide resistance gene *sul4*, macrolide resistance genes such as *macA/B, ermX,* diaminopyrimidine resistance gene *dfrA3,* fosfomycin resistance genes like *rosA/B, FosC2,* vancomycin resistance genes such as *vanI/TG/HM/HD/RE/RF*, lincosamide resistance gene *lmrC*, fluoroquinolone resistance genes *mupA/B*, and nitroimidazole resistance gene *msbA*. For Mb1, 5 resistance genes were predicted, including tetracycline resistance genes such as *tet(H), tetA (60)*, and *tet (35)*, the aminoglycoside resistance gene *vatB,* and the macrolide resistance gene *CRPm*1*el*. Both Pm1 and Mh1 contain multidrug efflux pump‐related genes, supporting their potential for multidrug resistance. In contrast, Mb1 has significantly fewer annotated resistance genes, primarily focused on tetracycline, aminoglycoside, and macrolide resistance genes.

## 4. Discussion

Bovine respiratory diseases are the most common diseases in the beef cattle breeding industry and have caused huge economic losses [17, 18]. It is mainly caused by the infections of Pm1, Mh1, and Mb1. Due to the diversity of pathogenic bacteria, the protective effects of existing vaccines are not satisfactory [19]. In this study, one strain each of type A Pm1, type A1 Mh1, and type Mb1 was sampled, isolated, and identified from dead cattle with bovine respiratory diseases. Subsequently, WGS and analysis of the isolated bacteria were carried out.

The genome size of Pm1 is similar to that of the first complete genome, Pm70 [20]. Through annotations in the COG, GO, and KEGG databases, it was found after analysis that the annotation results of these three databases were similar. The genes with high enrichment degrees were all involved in the metabolic processes of bacteria. Pm1 can obtain sufficient nutrients for growth and replication only through metabolic processes [21]. These metabolic processes also play a critical role in infection. Especially when the host immune system mounts an immune response, Pm1 can better absorb nutrients such as iron ions and amino acids to maintain basic metabolic survival only by changing the expression of genes involved in metabolic pathways [22, 23]; this metabolic adaptability is an important component of the virulence of Pm1. Therefore, vaccines developed against Pm70 may also provide protection against Pm1. Through analysis with the CAZy database, it was discovered that there were six types of carbohydrate‐active enzymes in the genome of Pm1, including glycosyltransferases and glycoside hydrolases. All 484 genes annotated in the PHI database have undergone phenotypic mutations, which play an important role in the research on finding target genes for drug intervention. The Pm1 genome has the most genes for the VFs LOS and capsule annotated by the VFDB, which is consistent with Pm1 of other serotypes [24]. Among them, LOS is a basic structural component on the cell wall of Gram‐negative bacteria, and its structure and biological functions are similar to those of LPS. It can trigger the host’s immune response by binding to Toll‐like receptors (TLRs) on host immune cells, such as macrophages and neutrophils, thereby inducing the release of proinflammatory cytokines (e.g., TNF‐α, IL‐1β, and IL‐6), leading to pulmonary inflammation and edema. The main difference is that LOS lacks O antigen. Among the 22 virulence genes detected in this study, only the *OmpA* and *fur* virulence genes were not annotated. Therefore, it is inferred that there may be certain false positives in the annotation results of VFs, and it is necessary to further verify their functions according to specific genes. The analysis of the CARD database shows that the Pm1 strain carries multiple resistance genes, although phenotypic resistance was not observed for all, suggesting possible transcriptional regulation or epigenetic mechanisms [25–28].

The results of the WGS of the isolate Mh1 showed that this strain carried a total of 233 virulence genes, 97 resistance genes, and 7 genomic islands. The resistance genes existed both in the genome and on plasmids. Among them, the resistance genes carried by plasmids included the diaminopyrimidine resistance gene *dfrA3* and the sulfonamide resistance gene *sul4*. The presence of these resistance genes is closely related to the strain’s drug resistance. Mh1 contains hemolysin genes *hlyA/B/C/D*, which can lyse mammalian red blood cells, releasing iron ions from hemoglobin for bacterial utilization. Hemolysins can also form pores in host cell membranes, leading to cell lysis and tissue damage, particularly causing severe inflammatory responses in the lungs [29]. In this study, a Type III secretion system (TTSS) was identified, which can directly inject virulence proteins into host cells, interfering with host signaling pathways and suppressing immune responses. Through TTSS, Mh1 can disrupt the normal functions of host cells, promoting bacterial colonization and dissemination. This system is a complex molecular apparatus found in many Gram‐negative pathogenic bacteria [30]. The identified Type IV secretion system (T4SS) may facilitate horizontal transfer of resistance and virulence genes [31, 32]. The resistance genes carried by the Mh1 strain (such as *dfrA3* and *sul4*) may spread among bacteria via plasmids, leading to the rapid dissemination of resistance [33, 34]. This spread of resistance exacerbates the difficulty of treating bovine respiratory disease and may contribute to more severe disease progression.

Genomic analysis of Mb1 revealed significant differences compared to the Canadian isolates [35]. When compared with the COG, GO, and KEGG databases, it was found that the enrichment degree of the metabolic process is the highest, indicating that Mb1 mainly obtains the main nutrients required for its survival through metabolic pathways [36, 37]. Through comparative analysis in the CAZy database, it was found that Mb1 has 8 genes related to auxiliary activities and 8 genes related to glycosyltransferases, which play important roles in the saccharide compounds and hydrolysis of organisms. In the CARD database, relatively few drug‐resistance genes were annotated, probably because they carry more virulence genes. Some studies have shown that there is a negative correlation between drug resistance and virulence [28]. Although Mb1 exhibited phenotypic resistance to enrofloxacin and carried quinolone resistance‐associated target genes such as *gyrA*, *gyrB*, *parC,* and *parE*, these genes were not annotated as confirmed resistance determinants in the CARD database. This may be because *gyrA*, *gyrB*, *parC*, and *parE* are conserved chromosomal target genes that are naturally present in bacteria, whereas fluoroquinolone resistance is mainly associated with mutations in the quinolone resistance‐determining regions (QRDRs) of these genes [38]. Since QRDR mutation analysis was not performed in the present study, the molecular basis of fluoroquinolone resistance in Mb1 requires further investigation. Similar findings have also been reported in previous studies, where phenotypic fluoroquinolone resistance could not be fully explained solely by CARD‐annotated acquired resistance genes [39]. After prediction by PHI, 125 genes of Mb1 were found to have mutated, which provides a basis for the possible emergence of new genes in the future [40]. The VFDB database was used to evaluate all the proteins of Mb1 to further explore the virulence‐related factors in pathogenicity. Mb1 adheres to host respiratory cells through the adhesion factor Vsp, avoiding clearance by ciliary movement and mucus. The adhesion factor also mediates the interaction between Mb1 and host cells, triggering cell damage and inflammatory responses [41]. Bacterial VFsare crucial for colonizing new habitats through infecting hosts and adapting to new environmental conditions [42, 43]. The *LplA* gene may be involved in lipid metabolism or host cell interactions in Mb1, but its specific functions still require further investigation. The contents and distributions of the adhesion, binding, transport, and protein synthesis genes of Mb1 are similar, and these genes may play important roles in adhering to and destroying host tissues, respectively [44, 45].

Several annotated virulence genes in Mb1, including cereulide (cesC) and cytolysin (cylB), are atypical for Mycoplasma and likely represent annotation artifacts. Read coverage and assembly quality did not support intact foreign genes, and no mobile genetic elements were identified adjacent to these loci. We therefore conclude these are false positives from VFDB annotation and should not be interpreted as genuine *M. bovis* VFs. This highlights the importance of manual curation for atypical findings in reduced‐genome bacteria [38, 39].

In the present study, a number of protein‐coding genes (512 in Pm1, 705 in Mh1, and 652 in Mb1) were not represented in the COG database. These unassigned genes most likely represent hypothetical proteins—genes predicted computationally but lacking functional annotation. This is common in bacterial genome sequencing and may also reflect the conservative bias of the COG database, which primarily includes genes with clear orthologs across multiple species. Additionally, a small proportion could represent assembly artifacts, although our use of hybrid sequencing (Illumina + Nanopore) minimizes this possibility. Genuinely strain‐specific unique genes may also be present, but functional validation (e.g., RNA‐seq or proteomics) would be required to confirm their expression and biological role. Through comparative analysis of the genomic characteristics, virulence, and resistance profiles of Pm1, Mh1, and Mb1, significant differences were observed in the distribution of functional genes, VFs, and drug resistance. Pm1 and Mh1 have larger genome sizes and a higher number of functional genes, particularly showing high gene enrichment in core functions such as amino acid transport and metabolism, carbohydrate metabolism, translation, and ribosome structure biogenesis. They also carry multiple resistance genes, indicating a high potential for multidrug resistance. In contrast, Mb1 has a smaller genome size and fewer functional and resistance genes, but it exhibits high enrichment in genes related to replication, recombination, and repair, which may be associated with its unique cell‐wall‐free structure and adaptability. In terms of virulence, Pm1 and Mh1 possess a higher number of virulence genes, and some genes show significantly enhanced virulence after mutation, indicating their strong pathogenic potential. Although Mb1 has fewer virulence genes, it still demonstrates enhanced virulence and chemical resistance after mutation, suggesting unique adaptive advantages in specific environments. Future research should further explore the mechanisms of identified virulence genes in host immune evasion and tissue damage, validate the regulatory mechanisms of resistance genes under antibiotic pressure, and reveal the adaptive evolution of pathogens by comparing the genomes of different strains. Based on these findings, the development of targeted vaccines and drug targets, particularly novel treatment strategies for multidrug‐resistant strains, will provide critical support for the prevention and control of bovine respiratory diseases.

## 5. Conclusions

In conclusion, this study delivers the first high‐resolution comparative genomic landscape of *P. multocida*, *M. haemolytica*, and *M. bovis*—the principal bacterial drivers of bovine respiratory disease. Our findings irrefutably establish that these pathogens employ fundamentally distinct genomic strategies: Pm1 and Mh1 rely on large, multidrug‐resistant genomes replete with diverse virulence arsenals, whereas Mb exploits a streamlined minimal genome optimized for immune evasion and persistence. Most importantly, the discovery of mobile genetic elements and biofilm‐associated genes across all three isolates unveils a previously underappreciated capacity for adaptation, resistance spread, and host immune counteraction. Collectively, these data do not merely describe genomic features—they provide a transformative foundation for next‐generation vaccines, rapid molecular diagnostics, and species‐tailored antimicrobial therapies. This work represents a critical step toward controlling bovine respiratory disease and reducing the multibillion‐dollar economic losses it inflicts on the global cattle industry.

## Author Contributions

Conceptualization, Hongxia Zhao; data curation, Yue Sun, Haiyan Dong, Wei Mao, and Hongxia Zhao; formal analysis, Yue Sun and Wei Mao; investigation, Kaiwen Yin, Haiyan Dong, Kaifan Han, and Weiguang Zhou; methodology, Kaiwen Yin, Shuying Liu, Yue Sun, Wei Mao, and Hongxia Zhao; resources, Kaifan Han; software, Shuying Liu and Weiguang Zhou; writing–original draft, Kaiwen Yin; writing–review and editing, Shuying Liu and Hongxia Zhao.

## Funding

This study was supported by funding from the Inner Mongolia Autonomous Region Science and Technology Major Project (Project No. 2021ZD0013); Inner Mongolia Autonomous Region First Class Discipline Research Special Project (Project No. YLXKZX‐NND‐012), China; and also supported by the Innovation Team of Education Department of Inner Mongolia Autonomous Region (NMGIRT2412).

## Ethics Statement

Current research was conducted according to the rules and regulations of the College of Veterinary Medicine (2022–2414), Inner Mongolia Agricultural University, Huhhot 010010, Inner Mongolia, China. All animal experiments, including bacterial challenge and LD50 determination, were approved by the Animal Ethics Committee of Inner Mongolia Agricultural University under approval number NND2022114.

Institutional Review Board Statement: The research was conducted after getting approval from the Laboratory Animal Welfare and Ethics Committee of Inner Mongolia Agricultural University, with ethical code NND2022114.

Informed consent was obtained and submitted in the journal.

## Consent

Please see the Ethics Statement.

## Conflicts of Interest

The authors declare no conflicts of interest.

## Data Availability

All data used in this article are available from the corresponding author upon reasonable request. For further information, please contact the corresponding author.
